# NF-KappaB in Long-Term Memory and Structural Plasticity in the Adult Mammalian Brain

**DOI:** 10.3389/fnmol.2015.00069

**Published:** 2015-11-24

**Authors:** Barbara Kaltschmidt, Christian Kaltschmidt

**Affiliations:** ^1^Molecular Neurobiology, University of BielefeldBielefeld, Germany; ^2^Cell Biology, University of BielefeldBielefeld, Germany

**Keywords:** NF-kappaB, long-term memory, transcription factors, synapse, dentate gyrus, long term potentiation

## Abstract

The transcription factor nuclear factor kappaB (NF-κB) is a well-known regulator of inflammation, stress, and immune responses as well as cell survival. In the nervous system, NF-κB is one of the crucial components in the molecular switch that converts short- to long-term memory—a process that requires *de novo* gene expression. Here, the researches published on NF-κB and downstream target genes in mammals will be reviewed, which are necessary for structural plasticity and long-term memory, both under normal and pathological conditions in the brain. Genetic evidence has revealed that NF-κB regulates neuroprotection, neuronal transmission, and long-term memory. In addition, after genetic ablation of all NF-κB subunits, a severe defect in hippocampal adult neurogenesis was observed during aging. Proliferation of neural precursors is increased; however, axon outgrowth, synaptogenesis, and tissue homeostasis of the dentate gyrus are hampered. In this process, the NF-κB target gene PKAcat and other downstream target genes such as Igf2 are critically involved. Therefore, NF-κB activity seems to be crucial in regulating structural plasticity and replenishment of granule cells within the hippocampus throughout the life. In addition to the function of NF-κB in neurons, we will discuss on a neuroinflammatory role of the transcription factor in glia. Finally, a model for NF-κB homeostasis on the molecular level is presented, in order to explain seemingly the contradictory, the friend or foe, role of NF-κB in the nervous system.

## What is memory?

In its simplest form, we can define memory as information that can be retrieved later when required. The frequently used definitions of memory in psychology are called declarative memory and non-declarative memory. These terms can be used to discriminate memories of tasks such as riding a bicycle (non-declarative) from other tasks such as remembering a lecture, events, or places (declarative). As one might see with this simple example, there is no easy way to describe how information from the real world (outer world) is stored within the brain. Current knowledge describes what is necessary and sufficient for long-term memory formation such as neurons, action potentials, synapses, engram cells, and neuronal networks. The term ‘engram’ was coined by Richard Wolfgang Semon, who was the first to call memory traces as engram (Semon, 1920). There are several hypotheses involving single synapses, such as the synaptic tagging and capture hypothesis (Redondo and Morris, 2011), or involving engram cells and their plasticity in neuronal networks (Ryan et al., 2015 and see below). It seems to be unclear that which information of the real world is stored and how so many different memories acquired during lifetime can be stored and retrieved. A standard approach to memory research is that the observation, i.e., the information from the real world, is taken up by sensory organs, e.g., eyes, and then the information is transformed into signals, which are transmitted to other neurons in the form of ion flux, generating action potentials (neuronal activity). Long lasting storage of this neuronal activity is thought to be stored in small subnets of neurons within the brain. These neurons fire together, show correlated responses, and have strong synaptic connections, which may be used for memory storage (see Mayford et al., 2012 for review), whereas most synapses, not integrated in the investigated subnet, have only weak connections. Kandel and coworkers provided another definition for memory: “Learning is the biological process of acquiring new knowledge about the world, and memory is the process of retaining and reconstructing that knowledge over time” (Kandel et al., 2014), suggesting that memory retrieval could alter memory itself. For additional details on electrophysiology and memory, see Section Target Genes with Potential Involvement in Memory.

Pioneering work of Eric Kandel and coworkers has led to the identification of molecular pathways that translate changes of gene expression to behavior. This process involves intracellular protein phosphorylation and later on the nuclear transcription factor CREB as a target of the protein kinase A (Kandel, 2001). These findings inspired the interest of investigating transcription factors as regulators of memory traces, taking up an old concept from Richard Semon, who called memory traces engram (Semon, 1920): “Ich bezeichne diese Wirkung der Reize als ihre engraphische Wirkung, weil sie sich in die organische Substanz sozusagen eingräbt oder einschreibt. Die so bewirkte Veränderung der organsichen Substanz bezeichne ich als das Engramm des betreffenden Reizes…” Own translation to English: “I call this action of a stimulus an engraphic action, because it engraves or writes into the organic substance. This change in the organic substance is called engram of the corresponding stimulus.” Recently Susumo Tonegawa and coworkers identified engram cells that retain memory even under retrograde amnesia (Ryan et al., 2015). They labeled engram cells by transcription of the activity-dependent *c-fos* promoter, driving tTA-dependent expression of either mCherry or chanelrhodopsin. Engram cells showed vast changes in synaptic plasticity on many spines. This could be fully inhibited by inhibiting protein synthesis. For an in depth discussion of the engram concept, see Ramirez et al. (2014). This work underscores the importance of transcription factors such as c-FOS for the formation of long-term memory in specific neurons (engram cells). We conclude that long-term memory could be stored in specific neurons (engram cells) and that engram cells are tagged during memory acquisition by the activation of transcription factors.

## Transcription factors and memory

As discussed previously, transcription factors might provide a way to measure memory traces in neuronal networks, which are involved in memory storage. What is the definition of a transcription factor? A recent census of human transcription factors was done by identifying proteins that bind DNA in a sequence-specific manner (Vaquerizas et al., 2009). For this analysis, DNA-binding domains and families from the Interpro database were assembled and used for database search. This led to the identification of 1391 transcription factors in the human genome. However, only 62 of these putative transcription factors have been experimentally verified for both DNA-binding and regulatory functions. The authors reported among the most-cited transcription factors such as p53, FOS, JUN, CREB, and NF-kappaB. Our own PubMed search in 2015 with MeSH entries “p53 and brain” resulted in 721 publications, whereas inclusion of “memory” in this search resulted in only 29 publications. Another PubMed search with “FOS and brain” resulted in 1399 publications, and with the additional search term “memory,” 76 publications were found. About 3196 publications were found for “CREB and brain”; 796 hits were found with the addition of search term “memory.” These results might underscore the importance of publications by Noble Prize winner Eric Kandel, who advanced the work on transcription and memory, especially on CREB (Kandel, 2001). Still NF-κB with MeSH term “brain” gives 1430 publications, and together with “memory,” we received 108 publications. Reading of the abstracts immediately revealed that some important work was not found by these search strategies. Therefore, here, we will review the current literature on NF-κB and memory in mammalian systems. Besides NF-κB, other transcription factors activated by neuronal activity and involved in memory include cAMP response element-binding protein (CREB), CCAAT enhancer-binding protein (C/EBP), activating protein 1 (AP-1), and early growth response factor (Egr) (Alberini, 2009). Certainly there could be more.

## Introduction to NF-κB

**Figure 1:**
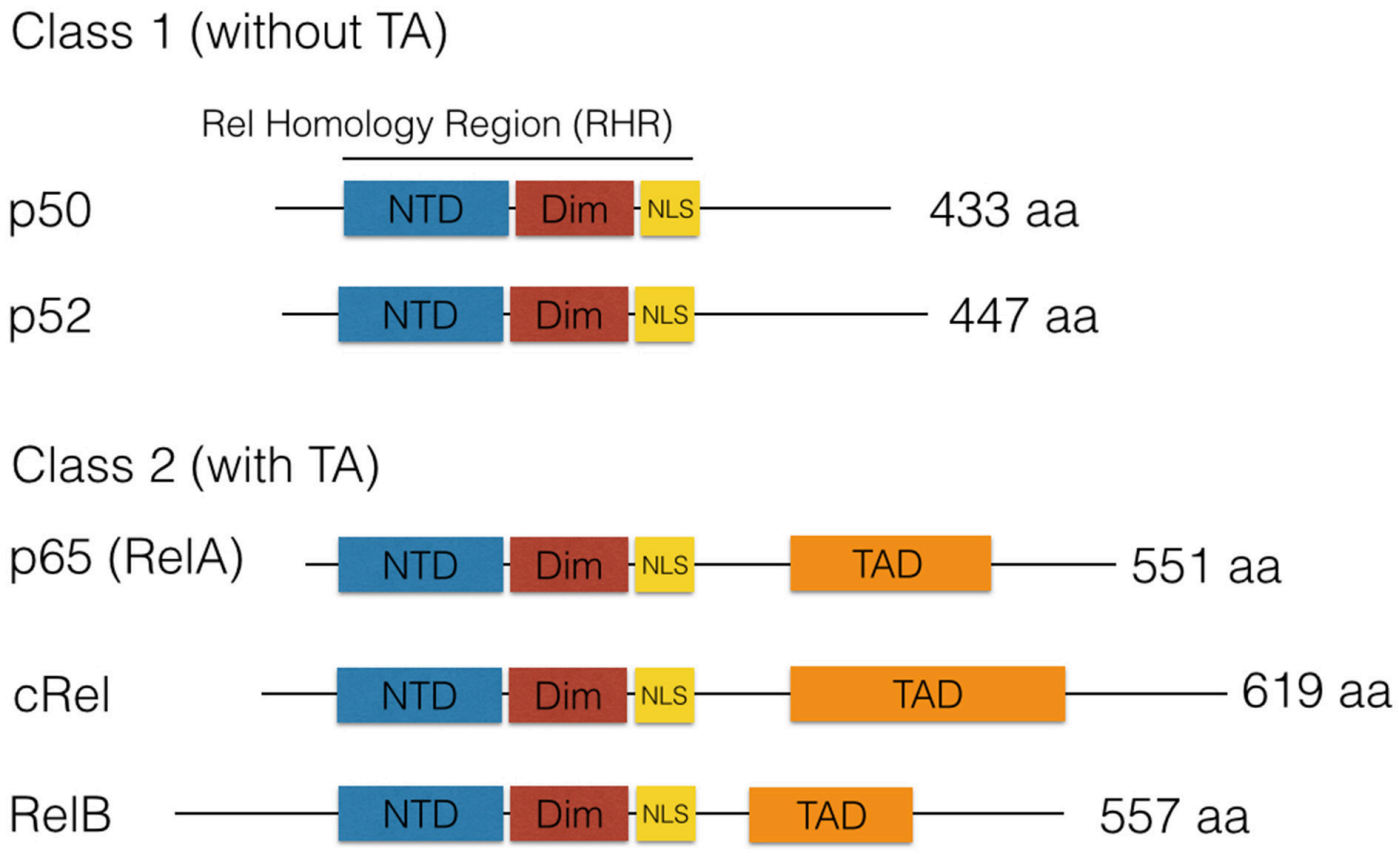
**The NF-κB family of DNA-binding proteins**. Five DNA-binding subunits found in the mammalian genome, which combine as homo- or heterodimers. A frequently found dimer is composed of p50/p65. All dimers can exist in a latent form in the cytoplasm complexed by IκB family members. This leads to inhibition of NF-κB due to cytoplasmic retention. Furthermore, NF-κB subunits can be classified as Class I (without transactivation, TA) or as Class II with TA due to the presence of a transactivation domain (TAD) in the C-terminal region.

NF-κB is a master transcription factor that is ubiquitously expressed and responds to diverse stimuli including cytokines, growth factors, and bacteria or viruses by the expression of stress response genes in many cells (Hayden and Ghosh, 2012). NF-κB (nuclear factor kappa B) was discovered in the laboratory of David Baltimore (Sen and Baltimore, 1986), as a DNA-binding factor that is specific to a 10-base pair nearly palindromic sequence: 5′-GGGACTTTCC-3, initially binding to the enhancer of the antibody kappa-light chain. Hence, this might explain its name. Cloning revealed a heterodimer composed of p50 kDa and p65 kDa (RelA) subunits. Further research identified other transcription factors with homology to the reticuloendotheliosis virus of turkeys (v-Rel). Each family member contains a Rel Homology region (RHR) near its N-terminus (Figure 1). The RHR contains two domains, the N-terminal domain (NTD) and the Dimerization domain (Dim), joined by flexible linker sequences. In addition, RHR contains a nuclear localization signal (NLS). Therefore, the RHR combines sequence-specific DNA-binding, dimerization, nuclear localization, and interaction with IκB proteins. IκB was purified by Patrick Baeuerle as a 60- to 70-kD inhibitory protein (called I kappa B) from a latent form of NF-κB in the cytoplasm, where latent NF-κB could be activated by treatment with mild detergent deoxycholate (Baeuerle and Baltimore, 1988a). These data suggested a non-covalent interaction of IκB with NF-κB, which could be purified and cloned (Baeuerle and Baltimore, 1988b). Later, many different proteins with IκB function were identified (Hinz et al., 2012).

## Activation of CREB and NF-κB in neurons

**Figure 2:**
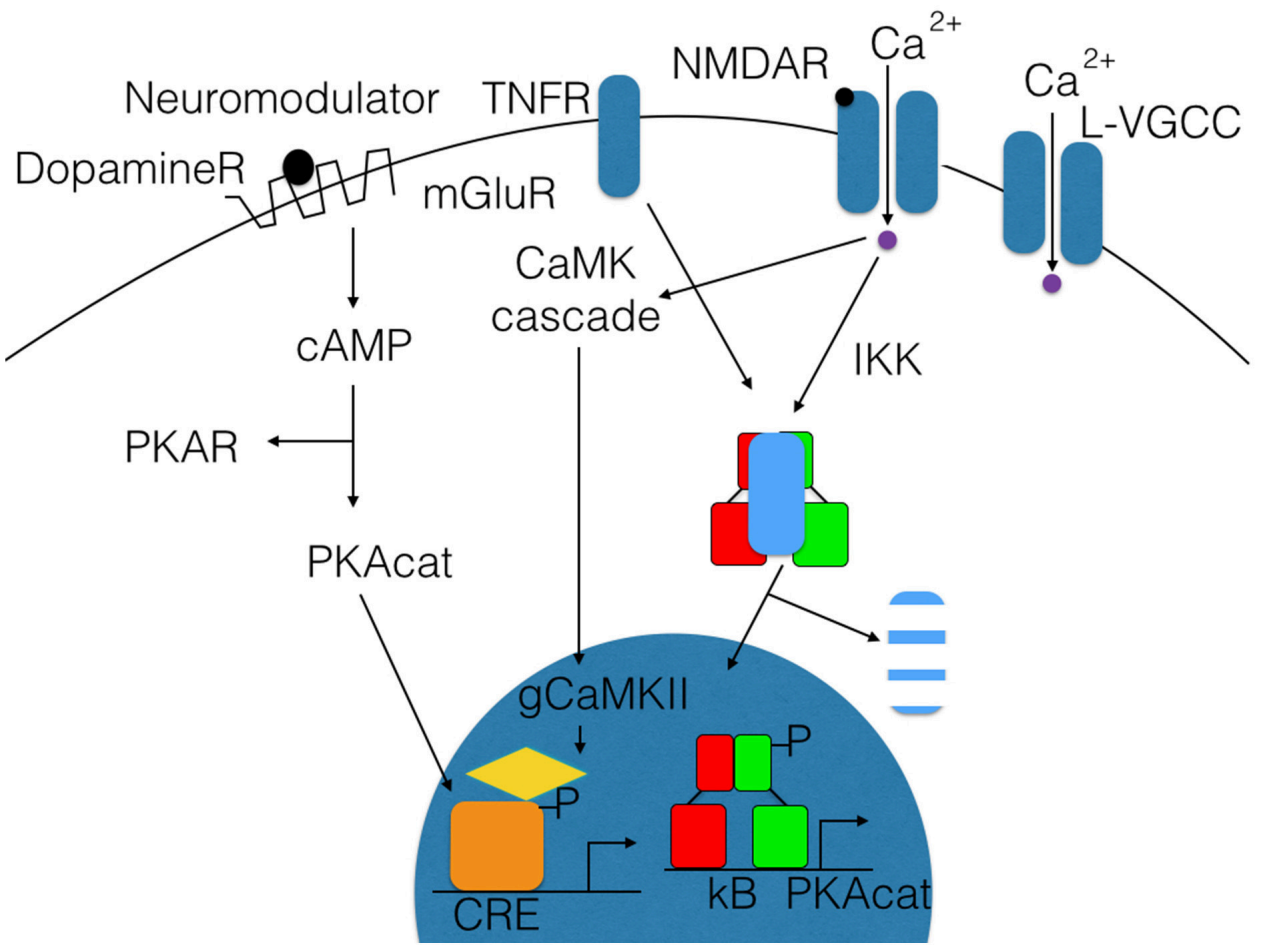
**Schematic representation for the activation of transcription factors like NF-κB and CREB by synaptic activity**. Upon synaptic stimulation of the receptors for NMDA or voltage-gated calcium channels (L-VGCC), a calcium influx induces activation of IkB kinases and phosphorylation of IkB (blue). Finally, degradation within the proteasome takes place, and thereby the nuclear localization signal of NF-kB is unmasked and allows NF-κB (red/green) to enter the nucleus, where it binds to kb sites and activates the transcription of target genes like PKAcat (catalytic subunit of protein kinase A). On the left site, CREB is activated after stimulation of mGluR or dopamine receptors by dopamine, and cAMP increases activating PKA. This phosphorylates the transcription factor CREB and other proteins like MAP kinases. P-CREB (orange) is bound by CBP (yellow), which initiates the transcription of its target genes. Interestingly, activation of NF-κB and expression of its target gene PKAcat will induce a positive feed-forward loop to CREB activation without additional synaptic activation, which might explain the late phase of long-term potentiation.

NF-κB is a transcription factor composed of two DNA-binding subunits that is activated by various stimuli in the nervous system (see Figure 2; Kaltschmidt and Kaltschmidt, 2009). Therefore, knockout or genetic ablation has various effects within the nervous system. In contrast, only knockout of subunit RelA (p65) is embryonically lethal. Genetic evidence for a role of NF-κB in the nervous system including cognitive effects or other neurological defects are summarized in Table 1.

**Table 1:** **Genetic mouse models interfering with NF-κB activity in the nervous system**.

**Genotype**	**Cell type afflicted**	**Cognitive effect**	**Additional phenotype**	**References**
p50^−∕−^	All	Defect in novel task acquisition; decreased anxiety; reduced short-term memory; Declining L-LTP; Egr-2 not upregulated after theta burst stimulation	Reduced neuroprotection; hearing loss; reduced neurogenesis; reduced ischemic damage; impaired acute and inflammatory nociception; Premature aging	Schneider et al., 1999; Yu et al., 1999, 2000; Kassed et al., 2002; Kassed and Herkenham, 2004; Duckworth et al., 2006; Niederberger et al., 2007; Denis-Donini et al., 2008; Oikawa et al., 2012; Bernal et al., 2014; Jurk et al., 2014; Nafez et al., 2015
P65^−∕−^	Isolated sensory neurons	na^*^	Reduced neuroprotection	Middleton et al., 2000
p65^−∕−^	Isolated Schwann cells	na^*^	Reduced myelination of peripheral nerves	Nickols et al., 2003
p65^−∕−^TnfrI^−∕−^	All	Delayed spatial learning in radial maze	No synaptic NF-kB	Meffert et al., 2003
CamKII tTA/tetO super-repressor IκB-α	Glutamatergic forebrain neurons	Impairments in spatial memory; reduced LTP and LTD; reduced spatial pattern separation; decreased synapse density (spine and presynapse); loss of mossy fibers	Reduced neuroprotection; decreased PKA expression and P-CREB; decreased neurogenesis; defects in dentate gyrus tissue homestasis	Fridmacher et al., 2003; Kaltschmidt et al., 2006; Imielski et al., 2012
Prion-tTA/tetO super-repressor IκB-α	Glutamatergic and inhibitory neurons	Enhanced spatial learning; enhanced LTD	Reduced GAD65 expression	O'Mahony et al., 2006
GFAP- super-repressor IκB-α	Glia: astrocytes	Deficits in learning only in females; delayed spatial learning, impaired cued fear memory	LTP reduced in females; LTP enhanced in males; reduction of mGluR5 in females; better recovery after spinal cord injury; reduced pain sensitivity	Brambilla et al., 2005; Bracchi-Ricard et al., 2008
c-Rel^−∕−^	All	Impaired late phase LTD; impaired long-term memory; impaired cued fear memory	Reduced neuroprotection; Late onset Parkinson	Pizzi et al., 2002; Levenson et al., 2004; Ahn et al., 2008; Baiguera et al., 2012
LsyM-Cre/IKK-2^FL∕FL^	Glia: microglia; macrophages	na^*^	30% reduction of neuronal death: 10-fold reduced infarct size after MCAO	Cho et al., 2008
Nestin-Cre/IKK-2^FL∕FL^	Precurosor and Neural (glia and neuron)	na^*^	25% reduction of infarct size after MCAO; amelioration of EAE	Herrmann et al., 2005; van Loo et al., 2006
Nestin-Cre/Ikk-1^FL∕FL^	Neural (glia and neuron)	na^*^	No effect on EAE	van Loo et al., 2006
Nestin-Cre/Nemo^FL∕FL^	Neural (glia and neuron)	na^*^	Amelioration of EAE	van Loo et al., 2006
NSE-SR-IκB-α	Neuronal	na^*^	Improved LPS-induced hypothermia and survival	Jüttler et al., 2007
CamK2a-tTA x luciferase-(tetO)7-IKK2-CA, called IKK2nCA	Neuronal	Defect in hippocampus-dependent learning and memory after 9 month measured in Morris water maze	Selective neuroinflammation; Reduction of BDNF expression; Granule cell degeneration	Maqbool et al., 2013
GFAP.tTA × (tetO)7.IKK2-CA	Astrocytes (Aldh1l1^+^)	Abnormal development of hippocampus and cerebellum; Strong upregulation of chemokines such as Ccl5, Cxcl10, and Ccl2, perhaps defect in neuronal stem cell migration	Defects could be induced during early development only: Hydrocephalus, massive increase of lateral ventricles, neuroinflammation; loss of cilia on ependymal cells in lateral ventricles	Lattke et al., 2012
Lentiviral constitutively-active IKKβ (^CA^IKKβ)	Mediobasal hypothalamus neurons	Reduced performance in T-maze	Life span decrease, GnRH expression reduced	Zhang et al., 2013
Nestin-Cre/IKK-2^FL∕FL^	Precurosor and Neural (glia and neuron)	Old mice perform better than WT in Morris water maze	Increased life span	Zhang et al., 2013

na^*^, not analyzed.

A comparison of NF-κB in the nervous system and CREB activation is shown in Figure 2. The CREB family contains CREB (cyclic-AMP-response element (CRE)-binding protein), CREM (CRE-modulatory protein), and ATF1 (activation transcription factor 1). Double-knockout of CREB and CREM resulted in a massive apoptotic cell death of neurons of the cortical plate and in the hippocampus and striatum, whereas neurogenesis seemed to be unaffected (Mantamadiotis et al., 2002), and no fiber defects were reported. Phosphorylation by protein kinase A at Ser133 activates CREB to promote transcription (for review, see West et al., 2002). Phosphorylation can lead to the recruitment of the transcriptional coactivator CREB-binding protein (CBP). CBP belongs to a family of histone acetyl-transferases, which catalyze hyper-acetylation of core histones, thereby leading to gene activation by loosening chromatin packing. Neuromodulators such as dopamine can activate G-protein-coupled receptors, which in turn activates membrane localized adenylate cyclase, which leads to the elevation of intracellular cAMP (Figure 2). Activation by cAMP frees the catalytically active protein kinase A (PKAcat) to enter the nucleus and phosphorylate CREB, which could ameliorate age-dependent memory deficits (Bach et al., 1999). Neuromodulators binding to metabotropic glutamate receptors can also activate NF-κB in neurons (O'Riordan et al., 2006). Activation of plasma membrane-localized ion channels, such as NMDA (*N*-methyl-D-aspartate) receptors (NMDARs) and L-type voltage-gated calcium channels (L-VGCCs), allow the influx of calcium (Figure 2). The classical view was that CREB could be activated by phosphorylation via the depolarization-activated Ca^(2+)^-calmodulin-dependent protein kinases (CaM kinases) I and II (Sheng et al., 1991). Then, how the calcium signal is transmitted to the nucleus? This story was unfolded about 25 years later. Recent results suggest a CaM kinase cascade (see Figure 2), where the calcium signal is transmitted via calmodulin-dependent phosphorylation of gamma CaMKII. The activated γCaMKII travels to the nucleus where phosphorylation of CREB is initiated (Ma et al., 2014). Until now, there are only two transcription factors known to decode frequency of calcium oscillations: NFAT and NF-κB (Dolmetsch et al., 1998). While NF-κB is already activated by low frequency of calcium oscillations and by infrequent oscillations even at the level of its target genes IL2 and IL8 (Dolmetsch et al., 1998), nothing is known about the situation in neurons. It is suggested that NF-κB could be activated by calcium in cerebellar granule cells (Lilienbaum and Israël, 2003). Further investigations might clear the mechanisms in other neuronal cell types. All NF-κB-activating stimuli culminate on the IKK complex composed from the IκB kinases IKK 1 and 2 (alpha and beta) and the third subunit NEMO (Israël, 2010). Activation of the IKK complex could involve ubiquitinylation of NEMO and upstream kinases, and the entire mechanism is not solved so far. Activation of the IKK complex leads to phosphorylation of the inhibitory subunit IκB alpha at Ser 32 and 36 followed by ubiquitinylation. This is a signal for proteolytic degradation of IκB in the proteasome. Anti-cancer drugs such as bortecomib (Velclade) are used to inhibit constitutive NF-κB activity in multiple myeloma plasmacytoma cells. In the context reviewed here, bortecimib can have serious side effects on the nervous system (see Velcade EMA/27714/2015) such as posterior reversible encephalopathy syndrome, autonomic neuropathy (damage to nerves controlling organs such as the bladder, eyes, gut, heart, and blood vessels), or more commonly peripheral neuropathy (nerve damage in hands and feet). Furthermore, inhibition of the proteasome system during consolidation blocked long-term memory both in crabs and mice (Sol Fustiñana et al., 2014). Degradation of IκB frees DNA-binding subunits to enter the nucleus by active transport (Figure 2), leading to the expression of target genes such as PRKACA (catalytic subunit of PKA; Kaltschmidt et al., 2006). NF-κB could be activated in neurons by depolarization, kainate, and glutamate (Kaltschmidt et al., 1995; Simpson and Morris, 1999). Stimulation in young cerebellar granule cells functions even at the concentration of 10 nM glutamate, alone or together with 10 μM glycine or 100 μM NMDA (Guerrini et al., 1995); interestingly, neurons prepared from older age depicted constitutive NF-κB activity as already reported for hippocampal and cortical neurons *in vitro* and *in vivo* (Kaltschmidt et al., 1994). This constitutive NF-κB activity could be modulated by co-cultured astrocytes, which could inhibit activation of NF-κB *in vitro* (Kaltschmidt and Kaltschmidt, 2000). Constitutive NF-κB activity is also found *in vivo* when reporter genes were analyzed. LacZ expression driven by NF-κB promoters (p105lacZ and 3 × Igk conalacZ) in brain was constitutive in various brain regions of the adult brain including cerebellar granule cells, the hippocampus and several layers as CA1, CA3 and dentate gyrus, and the cerebral cortex (Schmidt-Ullrich et al., 1996). Expression of reporter genes was only neuronal. Furthermore, forced expression of transdominant IκB alpha completely blocked constitutive expression of this lacZ NF-κB reporter gene (Fridmacher et al., 2003). Similar results were achieved with another mouse model (Bhakar et al., 2002) using a κB tandem repeat derived from HIV LTR to drive an SV40 minimal promoter lacZ gene. LacZ expression was strong in the telencephalon and along the roof plate of midbrain. Among other loci of staining, tactile hair follicles were prominently stained. Layers of the cortex cerebri are stained to a lesser extent, but strong staining was evident in hippocampal fields CA1, CA2, DG, and to a lesser extent in CA3. Forced expression of RelA protected cortical neurons against apoptosis induced by etoposide (Bhakar et al., 2002). We found that pre-conditioning neurons with tumor necrosis factor alpha (TNF) or low amounts of amyloid beta peptide (Aβ) could protect neurons against Aβ-mediated neurotoxicity (Kaltschmidt et al., 1999). In this line, it could be shown that TNF is involved in hippocampal synaptic plasticity (Albensi and Mattson, 2000). Furthermore, we have previously shown (Kaltschmidt et al., 1999) that treatment of cerebellar granule cells with TNF or low dose Aβ peptide (0.1 μM) could protect against neurotoxic amounts of Aβ (10 μM). This type of neuroprotection involves NF-κB and might be enhanced by CBP as a potential NF-κB target gene. CBP is a co-activator, which seems to be important for chromatin opening, due to its histone acetyltransferase activity (Ogryzko et al., 1996). Interestingly, several neuroprotective small molecules could be identified by a screening strategy using NF-κB activation (Manuvakhova et al., 2011). Memory decline is one of the hallmarks of Alzheimer's disease, which could be correlated to reduced NF-κB activity around Aβ containing plaques (Kaltschmidt et al., 1999). Neuroprotection against kainate was blocked by forced expression of transdominant IκB alpha (Fridmacher et al., 2003). In Figure 2, as an example of a target gene, the catalytic subunit of protein kinase A (PKAcat) is depicted; this gene is regulated by NF-κB in hippocampal neurons *in vivo* (Kaltschmidt et al., 2006). Forskolin-induced (cAMP-dependent) CREB phosphorylation is strongly impaired when NF-κB is repressed. These data suggest a cross-coupling of CREB and NF-κB-dependent memory pathways. However, the question which are the relevant memory target genes still remains.

## Target genes with potential involvement in memory

**Figure 3:**
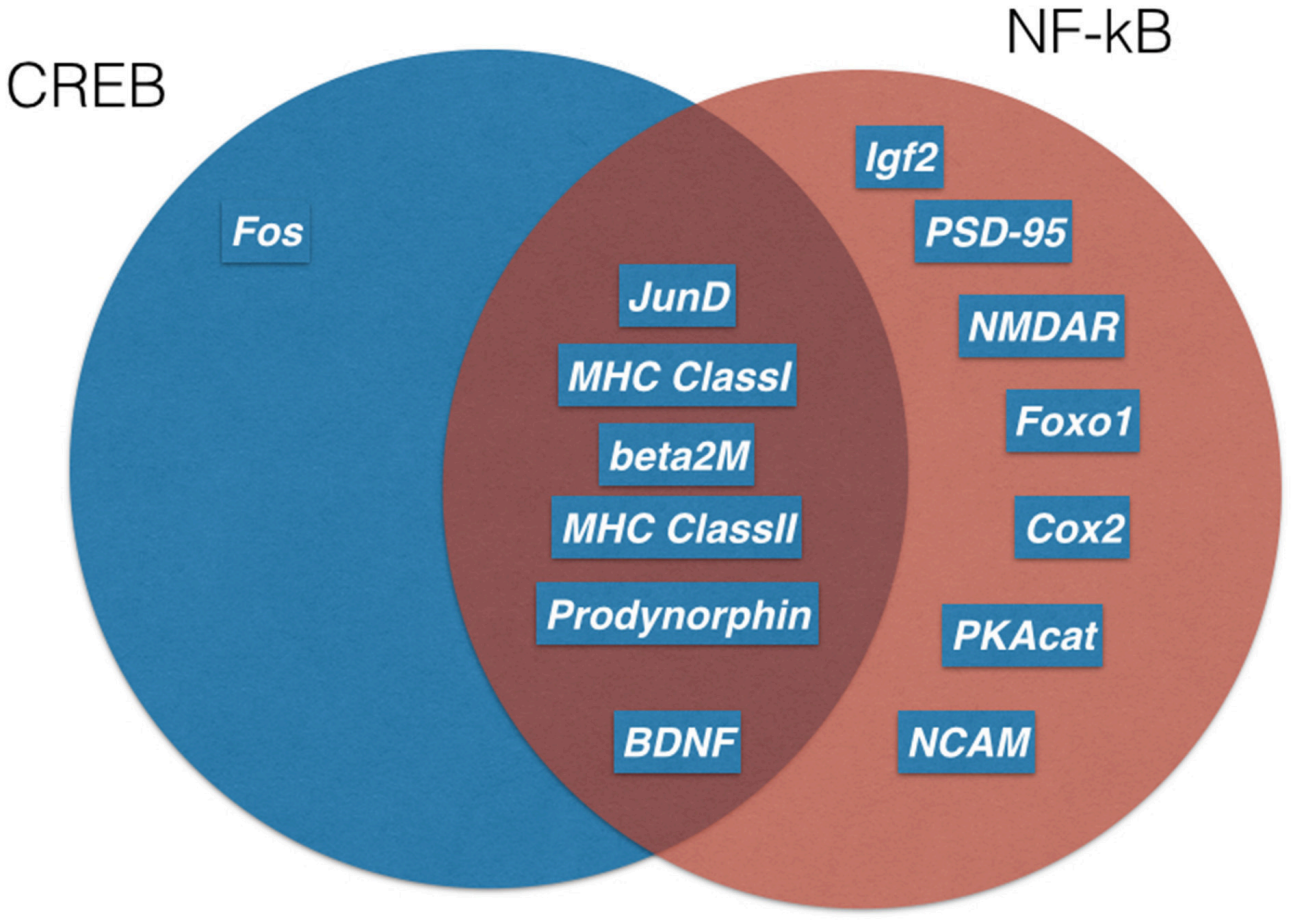
**Memory relevant target genes of NF-κB and CREB**. Genetic screens in transgenic mice identified several target genes of CREB (blue) and NF-kB(red). A Venn diagram shows overlapping target genes (dark red) of both transcription factors. For references see text.

**Figure 4:**
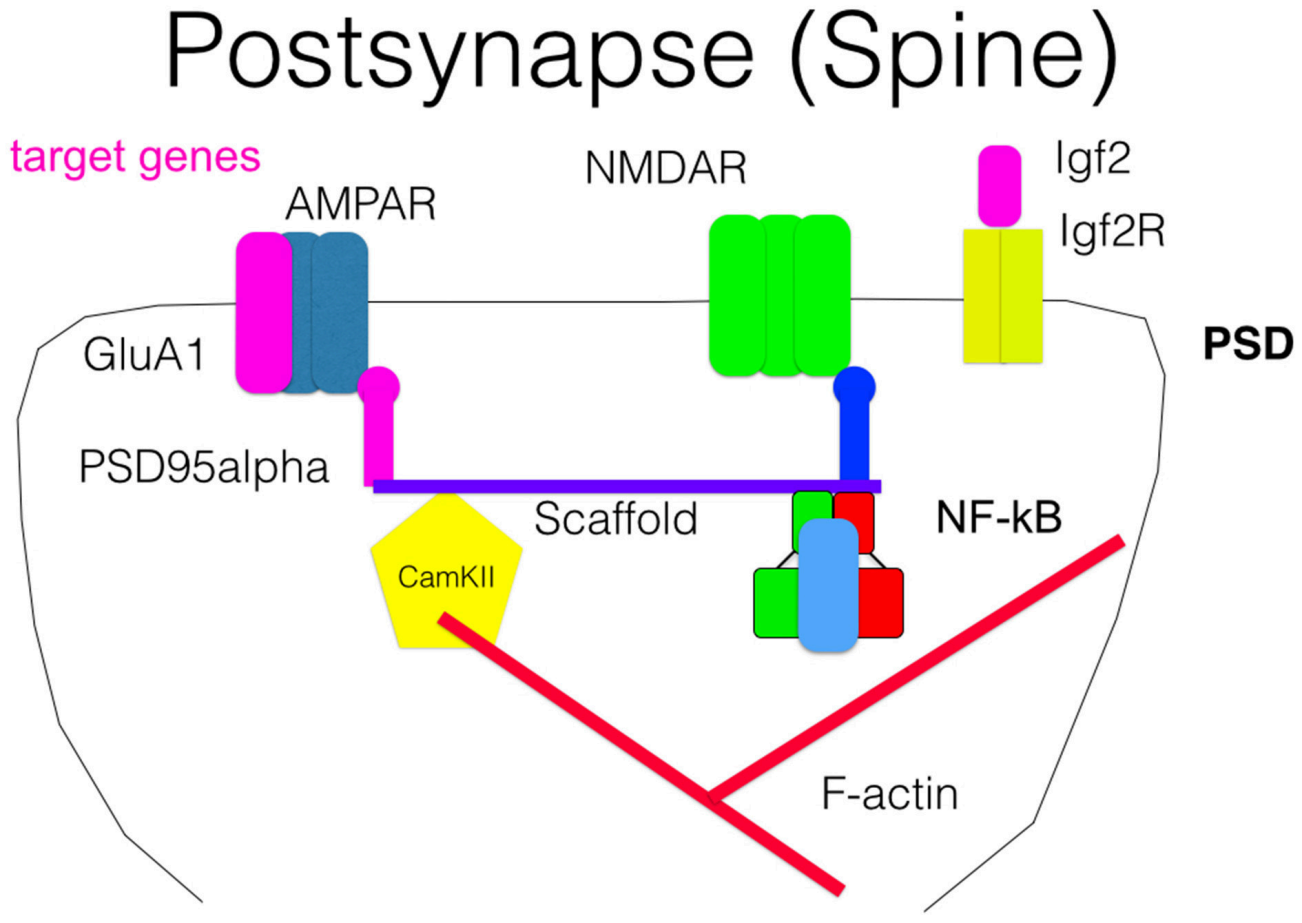
**Scheme of a post-synapse**. Some NF-κB target genes as AMPAR, Igf2, and PSD95 are crucial components of the post-synaptic compartment and involved in signal transmission. Association of NF-κB with the scaffold is suggested by biochemical fractionation but was not directly proven.

**Figure 5:**
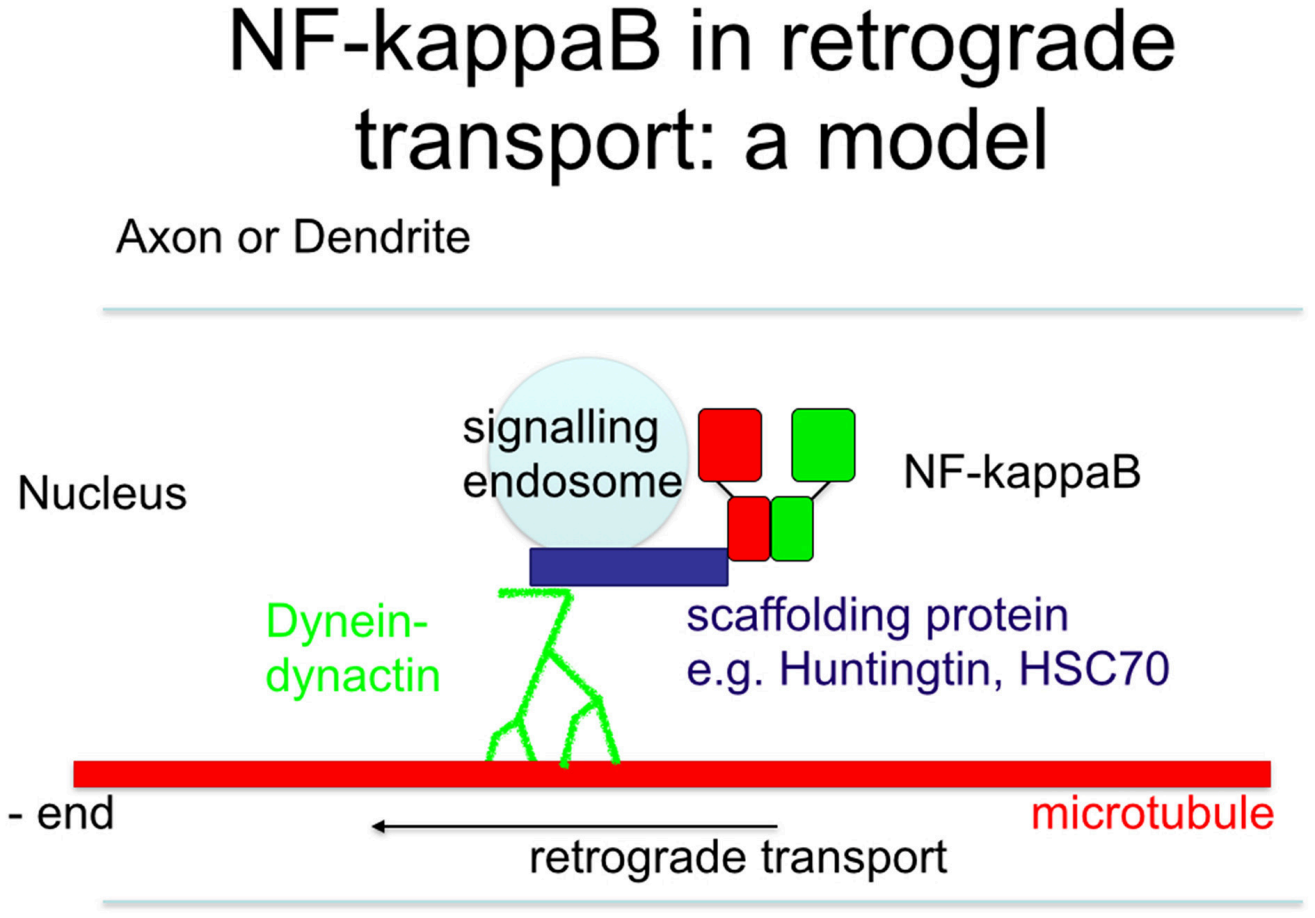
**Scheme of retrograde transport**. Upon stimulation of neurons, the nuclear localization signal is unmasked, allowing its binding to scaffolding proteins outside of the signaling endosome. This complex is then transported retrogradely to the nucleus, where it finally activates NF-κB target genes.

Long-lasting long-term potentiation (L-LTP) is a well-established model for memory at the synaptic level. In this paradigm, short voltage trains are used to induce a long-lasting post-synaptic response. It was discovered by Lømo when stimulating the perforant path (input to dentate gyrus granule cells) (Bliss and Lømo, 1973). Recording of field potentials of granule cells showed a long-lasting potentiation of granule cells lasting for hours (L-LTP). The requirement of L-LTP for protein synthesis was first described by Krug et al. (1984). *In vivo* L-LTP resulted in long-lasting biphasic CREB phosphorylation (Schulz et al., 1999). NF-κB p50^−∕−^ animals showed only decremental LTP that faded over time, even when high frequency stimulation trains were used, which elicited L-LTP in control animals (Oikawa et al., 2012). Furthermore, LTP magnitude and LTD induction were blocked by inhibiting NF-κB DNA binding in hippocampal slices (Albensi and Mattson, 2000). In this line, expression of Egr-2 was upregulated in WT hippocampal slices preparation after theta burst stimulation but not in p50^−∕−^ slices (Nafez et al., 2015). Same authors identified Egr-2 as novel NF-κB target gene in brain. Knockout of c-Rel also led to decremental LTP, whereas c-Rel expression was up-regulated during novel object recognition (Ahn et al., 2008). Late-onset Parkinson's disease was reported in mice with c-Rel knockout with loss of tyrosine hydroxylase positive neurons in the substantia nigra only visible at 18 month of age (Baiguera et al., 2012). Inhibition of all NF-κB subunits by forced IκB expression led to reduced LTP induction and longer learning phases in Morris water maze (Kaltschmidt et al., 2006). As potential mechanisms, the current models discuss the formation of novel post-synapses and NMDA receptor density increase. Previous reviews suggested that the two secreted proteins, brain-derived neurotrophic factor (BDNF) and tissue plasminogen activator (tPA), have been repeatedly implicated in L-LTP as memory-relevant CREB target genes (Kandel, 2001) or nNOS and presenilin as additional CREB targets (West et al., 2002). To analyze this question in more detail, Eric Kandel and coworkers expressed a constitutively transactivating mutant of CREB (VP16-CREB) in transgenic mice under the control of the tetracycline operator, which can be activated by CamKII promoter-driven expression of the tetracycline-dependent transactivator (Barco et al., 2005). Surprisingly, this study identified only a handful of genes that are activated by VP16-CREB-induced L-LTP, but not by increased neuronal activity alone (kainate treatment). These genes include prodynorphin, MHC Class I and JunD/cFos in CA1, and dentate gyrus (see Figure 3). A low amount of induction in all hippocampal subfields was detected for BDNF. Surprisingly, Arc was only induced by kainate treatment, suggesting a difference in genes activated by neuronal activity and memory-relevant gene expression. Furthermore, MHC Class II genes seemed to be induced by VP16-CREB. These data could be reproduced in part with a cellular model of cultured hippocampal neurons (Benito et al., 2011). One might be surprised by the fact that a similar set of genes were identified in a different but technically similar *in vivo* screen, involving loss of function of NF-κB (Kaltschmidt et al., 2006). Therefore, we suggest that JunD, MHC Class I, II and beta 2 microglobulin, prodynorphin, and BDNF might be a common set of genes that are regulated by CREB and NF-κB together. Indeed, MHC Class I, II and beta 2 microglobulin share common response elements including the CREB-binding site, CRE (Gobin et al., 2001). Similarly, these genes are NF-κB target genes (Israël et al., 1989) even in neurons (Kaltschmidt et al., 1995; Yang et al., 2014). However, function in neurons remained unclear. MHC Class I molecules are trimeric proteins that are composed of a transmembrane heavy chain, a soluble κ2-microglobulin (κ2-m) light chain, and a peptide bound to the heavy chain. Only recently, Carla Shatz and coworkers have suggested a synaptic glue hypothesis (Huh et al., 2000), where MHC Class I molecules might be used for adhesion of specific synapses. Surprisingly, LTP is enhanced and LTD is absent in mice without functional MHC Class I (Huh et al., 2000). Expression of MHC Class I genes H2-D and T22 could be detected in the hippocampus (Huh et al., 2000). However, other authors argue that MHC Class I has a function in pathological situations (e.g., synapses and neuron elimination by T-cells; Cebrián et al., 2014). Similarly, no memory-relevant target genes for the AP-1 complex (Fos/Jun) were presented so far. Prodynorphin was identified as a NF-κB target gene with mapped response sites (Bakalkin et al., 1994). BDNF expression might be regulated indirectly via the NF-κB target gene XIAP (Kairisalo et al., 2009). In our humble opinion, the correlation with memory formation is better for NF-κB target genes (Figure 3 right part of the Venn diagram). The initial idea of synaptic enhancement by transcription is of a target gene that can indeed enhance post- or pre-synapse function. We think there are now, after more than 20 years of research on NF-κB in the nervous system, enough evidences on involvement of NF-κB target genes in synaptic enhancement. In this line, a recent genetic screen identified an insulin-like growth factor 2 (Igf2, see Figure 4) as a novel neuronal NF-κB target gene involved in spine density regulation (Schmeisser et al., 2012). Interestingly, Igf2 could rescue the spine reduction in neurons with NF-κB inhibition (Schmeisser et al., 2012). In addition, Igf2 is critically involved in memory consolidation and enhancement (Chen et al., 2011). Igf2 could ameliorate the neurodegeneration in a mouse model with Alzheimer's disease, and its expression is reduced in Alzheimer patients' hippocampi (Pascual-Lucas et al., 2014). Similarly, NF-κB activity was reduced in Alzheimer patients' brains (Kaltschmidt et al., 1999), suggesting that Igf2 could be a target gene of NF-κB in humans. In addition, amyloid beta peptides could activate NF-κB p65 in neurons and might activate NF-κB in the close vicinity of early plaques in Alzheimer patients' brains (Kaltschmidt et al., 1997). In this respect, it might be important to note that the activation of NF-κB by Alzheimer's disease follows a bell-shaped curve where low amounts of Aβ activate NF-κB, but high amounts repress NF-κB; for discussion, see Kaltschmidt et al. (2005). In a recent study (Schmeisser et al., 2012), a dominant negative version of the IKK2 protein was expressed in neurons using the tet system. Spine density was reduced to the half of control in CA1, especially the memory-relevant mushroom spines were reduced to about 25%. Learning was slower as with IκB over-expression. Synapses contained significantly less GluA1 and PSD95. Ultrastructure depicted a high amount of synapses with disrupted post-synaptic density. Similarly, it was shown earlier in cultured neurons that NF-κB activity positively regulated spine densitiy and glutamatergic synapse formation (Boersma et al., 2011). These data describing post-synaptic damage fit to our data reporting reduced size and number of presynaptic mossy fiber buttons (Imielski et al., 2012). In addition, we observed that axonal growth is regulated by NF-κB *in vitro* and *in vivo* in the mossy fiber pathway (Imielski et al., 2012). Taken together, NF-κB regulates post-synapse formation (see Figure 4) by expression of Igf2, whose receptor is post-synaptic and by additional target genes such as PSD95 (Boersma et al., 2011). Furthermore, NF-κB directs the expression of AMPA receptor components (Boersma et al., 2011; Schmeisser et al., 2012). NMDA receptor component NR1 appears to be regulated by NF-κB, and regulation of NF-κB target genes such as Cox2 (Kaltschmidt et al., 2002), PKAcat (Kaltschmidt et al., 2006), Foxo1, and NCAM (Imielski et al., 2012) were described. Localization of NF-κB in synaptosomes (Kaltschmidt et al., 1993; Meberg et al., 1996; Meffert et al., 2003; Schmeisser et al., 2012) suggests a function of NF-κB as retrograde messenger (see Figure 5). In this line, we and others could show that p65-GFP is traveling to the nucleus when activated by glutamate (Wellmann et al., 2001; Meffert et al., 2003). This retrograde transport is dependent on the nuclear localization signal of p65 and involves a dynein–dynactin motor protein complex traveling on microtubules (Mikenberg et al., 2007; Shrum et al., 2009). The traveling complex seems to be a signaling endosome with scaffolding proteins such as Huntingtin and/or HSC70 (Marcora and Kennedy, 2010; Klenke et al., 2013). In comparison, it might be interesting to note that CREB could be translated in axons and mediate a retrograde survival signaling in response to nerve growth factor (Cox et al., 2008).

## NF-κB signaling is essential for dentate gyrus tissue homeostasis

**Figure 6:**
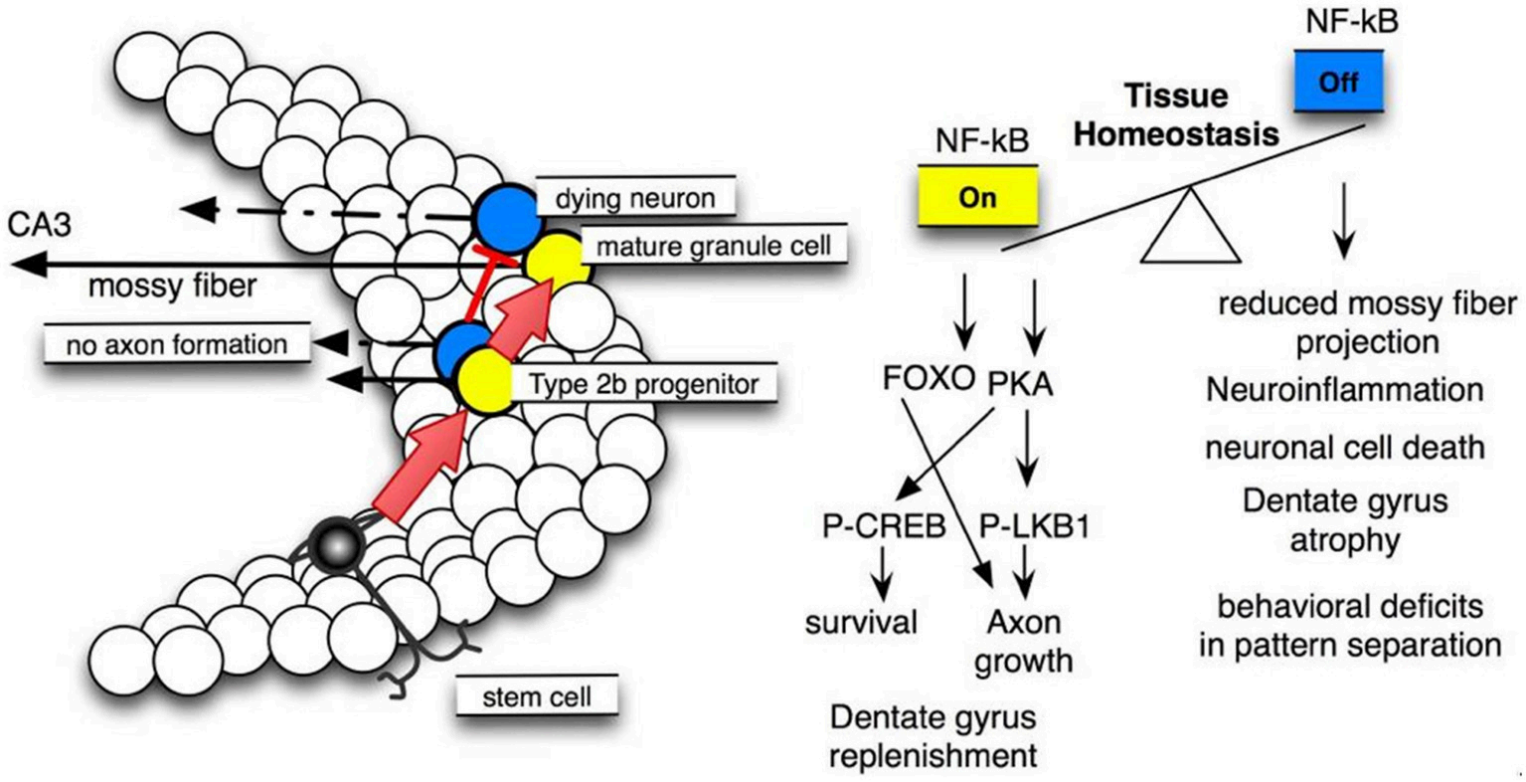
**NF-κB signaling is crucial for dentate gyrus tissue homeostasis**. **Left site:** scheme of neural stem cell differentiation in the dentate gyrus, note that NF-κB activation is essential for type 2b progenitor (yellow) maturation to mature granule cells. **Right site:** molecular pathways involved and resulting phenotypes.

Previously, we have shown that NF-κB plays an important role at three consecutive stages of neurogenesis (see Figure 6): proliferation/differentiation of neural progenitor cells, axon specification, and integration of young neurons and survival of mature granule cells (Imielski et al., 2012). Recently, the role of NF-κB p50 in neurogenesis was analyzed (Denis-Donini et al., 2008) in p50^−∕−^ mice, where the net rate of neural precursor proliferation was unchanged, but only 50% of newborn neurons survived in the DG and a defect in spatial short-term memory was observed. Because in p50^−∕−^ mice only one NF-κB subunit is deleted in all cell types, we used (Imielski et al., 2012) a neuronal-specific ablation of all NF-κB subunits in our study. We confirmed in part the results of Denis-Donini and coworkers for a role of NF-κB in neurogenesis and extended that findings to a function of NF-κB in tissue of the dentate gyrus homeostasis, synaptogenesis, axogenesis, and spatial pattern separation. In contrast to Denis-Donini et al., we found an increased rate of proliferating DCX-positive granule cell progenitors, presumably due to the high rate of apoptosis observed in DG after forced expression of transdominant negative IκB-alpha. Regrowing of DG after re-activation of NF-κB had a major impact on integration and survival of newborn DCX+ neurons. Similarly, neurogenesis within the subventricular zone depends on NF-κB (Zhang et al., 2012).

The regulation of adult neurogenesis by transcription factors is still a matter of debate (Chen et al., 2011), and our data show that the transcription factor NF-κB is a crucial regulator of neurogenesis, essential for axogenesis, and integration of newborn neurons. Taken together, these data indicate that NF-κB plays an important role in structural plasticity of the hippocampus.

Surprisingly, another study showed that over-activation of NF-κB in a transgenic mouse model with forced expression of ncaIKK-2 (Maqbool et al., 2013) resulted in a similar structural defect: degeneration of granule cells to about 50% of control level and massive astrocytosis. In addition, a reduction of BDNF was described but no decrease of Prkca was measured. However, expression of the NF-κB target gene Igf2 was not elevated in this mouse model, arguing against the validity of gain of function approaches to study NF-κB target genes.

To the best of our knowledge, NF-κB is the one and only factor where ablation or over-activation showed such severe effects in structural plasticity.

The observed NF-κB-dependent structural defects resulted in a behavioral phenotype. Recently, behavioral tests (Clelland et al., 2009) were developed to measure the memory of subtle differences in spatial environment. The authors suggest that neurogenesis enhanced the recognition of weakly separated cues. In this line, we developed a special behavioral test (*SPS-BM*), which is able to measure spatial pattern separation with the advantage to analyze search strategies (Widera et al., 2014).

Interestingly, the mouse model described in Imielski et al. (2012) is a phenocopy of Alzheimer's disease (AD) in three aspects: reduced NF-κB activity as in AD brains (Kaltschmidt et al., 1999), increased proliferation of immature DCX+ neuronal precursors, and progressive cell loss (Jin et al., 2004) coupled with strong neuroinflammation. Thus, re-activation of NF-κB might be an interesting therapeutic strategy for neuroregeneration of the adult dentate gyrus in the future.

## Conclusion

We suggest that NF-κB activation in neurons could be an interesting strategy to ameliorate memory diseases such as Alzheimer's disease. Dose–response curve of such kind of drugs might be bell shaped, too much will lead to neurodegeneration. Furthermore, constitutive activation of NF-κB in glia during aging might lead to cognitive decline. Finally, re-activation of NF-κB inhibition might lead to neuroregeneration due to stem cell action, which could be the basis for further understanding of brain repair.

### Conflict of interest statement

The authors declare that the research was conducted in the absence of any commercial or financial relationships that could be construed as a potential conflict of interest.
